# Directly observed and reported respectful maternity care received during childbirth in public health facilities, Ibadan Metropolis, Nigeria

**DOI:** 10.1371/journal.pone.0276346

**Published:** 2022-10-21

**Authors:** Oluwaseun Taiwo Esan, Salome Maswime, Duane Blaauw

**Affiliations:** 1 Department of Community Health, Faculty of Clinical Sciences, Obafemi Awolowo University, Ile-Ife, Osun State, Nigeria; 2 Centre for Health Policy, School of Public Health, Faculty of Health Sciences, University of the Witwatersrand, Johannesburg, Gauteng, South Africa; 3 Global Surgery Division, Department of Surgery, Faculty of Health Sciences, University of Cape Town, Cape Town, Western Cape, South Africa; Flinders University, AUSTRALIA

## Abstract

Respectful maternity care (RMC) is believed to improve women’s childbirth experience and increase health facility delivery. Unfortunately, few women in low- and middle-income countries experience RMC. Patient surveys and independent observations have been used to evaluate RMC, though seldom together. In this study, we assessed RMC received by women using two methodologies and evaluated the associated factors of RMC received. This was a cross-sectional study conducted in nine public health facilities in Ibadan, a large metropolis in Nigeria. We selected 269 pregnant women by cluster sampling. External clinical observers observed them during childbirth using the 29-item Maternal and Child Health Integrated Program RMC observational checklist. The same women were interviewed postpartum using the 15-item RMC scale for self-reported RMC. We analysed total RMC scores and RMC sub-category scores for each tool. All scores were converted to a percentage of the maximum possible to facilitate comparison. Correlation and agreement between the observed and reported RMC scores were determined using Pearson’s correlation and Bland-Altman analysis respectively. Multiple linear regression was used to identify factors associated with observed RMC. No woman received 100% of the observed RMC items. Self-reported RMC scores were much higher than those observed. The two measures were weakly positively correlated (rho = 0.164, 95%CI: 0.045–0.278, p = 0.007), but had poor agreement. The lowest scoring sub-categories of observed RMC were information and consent (14.0%), then privacy (28.0%). Twenty-eight percent of women (95%CI: 23.0% -33.0%) were observed to be hit during labour and only 8.2% (95%CI: 4.0%-18.0%) received pain relief. Equitable care was the highest sub-category for both observed and reported RMC. Being employed and having completed post-secondary education were significantly associated with higher observed RMC scores. There were also significant facility differences in observed RMC. In conclusion, the women reported higher levels of RMC than were observed indicating that these two methodologies to evaluate RMC give very different results. More consensus and standardisation are required in determining the cut-offs to quantify the proportion of women receiving RMC. The low levels of RMC observed in the study require attention, and it is important to ensure that women are treated equitably, irrespective of personal characteristics or facility context.

## Background

Sub-Saharan Africa, with a maternal mortality ratio (MMR) of 542/100,000 live births, accounted for two-thirds of global maternal deaths in 2017 [1]. Skilled birth attendance (SBA) could reduce maternal deaths by 13–33% [2]. However, only 59% of deliveries in sub-Saharan Africa were attended by skilled birth attendants between 2009 to 2018 [3]. Nigeria had the 4th highest MMR in Africa in 2017, based on modelled estimates [4]. Nigeria’s MMR for women aged 15–49 years was 512 per 100,000 live births between 2012 and 2018, with a lifetime risk of maternal death of 1 in 34 before the age of 50 [5]. Only 39.4% of Nigerian women delivered at a health facility between 2013 and 2018, and this indicator decreases to only 11.6% amongst women in the lowest wealth quintile [5]. For Africa to attain the Sustainable Development Goal (SDG) 3 target of reducing MMR to below 70 per 100,000 live births, significant improvements in maternal health are required in countries with the highest maternal mortality indices. This includes Nigeria, one of the largest countries in the region.

Negative health worker attitudes expressed as mistreatment during childbirth are known deterrents to health facility delivery [6]. A high global burden of mistreatment of women during childbirth has been reported, particularly in low- and middle-income countries, (LMICs) [6]. The burden of women’s mistreatment during childbirth in Nigeria ranged between 11% and 71% in a review of published papers between 2004 and 2015 [7].

Respectful maternity care has been proposed as a strategy aimed to give women a positive childbirth experience. Respectful maternity care (RMC) during childbirth is care without physical or verbal abuse, delivered with dignity and respect for women’s preferences, providing adequate information and obtaining informed consent before procedures [8, 9]. It is also equitable care delivered regardless of the women’s characteristics by competent and motivated staff who are available when needed, who do not neglect their clients, and who communicate effectively without language barriers. It entails the provision of quality and effective professional treatment in a comfortable, clean and calming environment that ensures privacy and continuous access to birth companions. In delivering RMC, the women’s choices for mobility during labour, intake of fluids or oral foods and alternative birth position should also be respected [9]. The World Health Organisation (WHO) had declared RMC as the standard of care for all women [10]. Unfortunately, receipt of RMC is still often seen as a luxury, with few women in LMICs currently accessing it [11], despite the recent global efforts, research and interventions to promote RMC [12, 13].

To support the planning of RMC-promoting interventions in LMICs, it is necessary to measure to what extent RMC is being received, who receives it, and which components are received. This raises the challenge of how to measure RMC [14]. Client or provider interviews are most often reported in the literature [15–18]. However, there have been calls recommending a mixed-method approach, including independent observations to measure RMC [13, 19, 20]. There are few documented RMC observational studies from Nigeria [21, 22]. Observational studies are often challenged by the possibility of a Hawthorne effect, the ethics of observing women during labour and the cost of conducting the research compared to a client interview. Our study measured RMC received during childbirth using both observation and structured interviews across two levels of healthcare delivery and evaluated the factors associated with the observed RMC scores. We also compared the level of agreement between both methodologies.

## Methods

### Study design and setting

This was a cross-sectional study conducted from November 1, 2019, to March 31, 2020. We observed women during childbirth and interviewed the same women more than 12 hours postpartum to evaluate the RMC received during childbirth. The study was conducted in public health facilities across the five local government areas (LGAs) in Ibadan Metropolis. Ibadan is the third largest city in Nigeria and the seventh in Africa [23] The LGAs are Ibadan North, North-East, North-West, South-East and South-West.

### Sampling technique and sample size

Primary and secondary health facilities across the five LGAs with more than 1 delivery per month were identified from the state health information system. This gave a sampling frame of 6 secondary and 26 primary health facilities. A two-stage cluster sampling was done to select the study health facilities and the respondents. One primary and one secondary health facility were selected from each LGA using simple random sampling except for the South-East LGA which had no secondary health facility. This gave a total of nine health facilities. All consenting women arriving in labour at any time of the day during the observation period (1 month) per facility were to be observed.

A minimum sample size of 258 was calculated using the one sample proportion test in Stata [24] based on a reference proportion of 81% for women who did not experience disrespectful and abusive care during childbirth but received RMC in a study in Ile-Ife, Nigeria measured through client interviews [25]. Other parameters used were a ±10% difference around the reference proportion, 90% power, and a design effect of 2 for the cluster sampling [26]. However, to get a final sample of 258 women who had been both observed and interviewed postpartum for analysis, we estimated a 20% attrition rate between observation and interview to account for any women referred out during or after labour who would then not be available for interview at postpartum. This gave a total sample of 322 women to be observed.

### Recruitment

A research assistant recruited booked pregnant women in their third trimester of pregnancy from those attending the antenatal clinic (ANC) at each of the study facilities. This was done a month before the scheduled data collection period at each facility. Written informed consent was obtained for both the observational and interview components of the study. Consent was preferentially done at ANC to avoid obtaining consent from the women while in labour, as they may be more vulnerable at this time [27]. However, the observers also obtained consent from unbooked women presenting in labour. Unbooked women were included to ascertain the effect of booking status on their childbirth experience. A tag with the name of the project was attached to each consenting woman’s hospital file to enable identification during labour.

### Observation of RMC received during childbirth

A total of 322 women were observed during childbirth. We used the adapted MCHIP RMC standard checklist by Jhpiego [28], which has 29 items describing 7 categories of mistreatment based on the Bowser and Hills criteria [29]. The observed RMC sub-categories are presented in Table 1. The variables ‘welcomed/greeted the woman’ and obtained consent/permission were added to the information and consent sub-category to make 11 items. This was because how women were greeted on presentation in labour was reported as important in an earlier phase of the study that explored women’s perceptions of RMC during childbirth [30]. Also, the original MCHIP tool combined the provision of information before procedures and obtaining consent in the same question. These were split these because we believed that providing information and obtaining consent were not synonymous. The 7th sub-category ‘assessing the facility’s policy on illegal detention of women unable to pay the bills’ was dropped as it could not be evaluated during the observation. The reliability measures for the tools are shown in Table 1. Three of the observed RMC sub-scales and one for reported RMC had Cronbach’s alpha <0.5. The Cronbach’s alpha for the overall observed and reported RMC scales were 0.685 and 0.820 respectively.

**Table 1:** Breakdown of sub-categories for the observed and reported RMC tools.

	Observed RMC sub-categories	Items	Alphapha		Reported RMC sub-categories	Items	Alpha
1	Dignified and respectful care	3	0.283	1	Friendly care	7	0.865
2	Protection from physical harm	6	0.548	2	Abuse free care	3	0.579
3	Non-abandonment/neglect	3	0.674	3	Timely care	3	0.397
4	Equitable care	2	0.588	4	Non-discriminatory care	2	0.655
5	Care in privacy and confidentiality*	4	0.104				
6	Information and informed consent*	11	0.332				
	**Overall observed RMC tool**	**29**	**0.685**		**Overall reported RMC tool**	**15**	**0.820**

The asterisked sub-categories had no related sub-category in the reported RMC tool

Two groups of three recently-qualified registered nurses conducted the observations as external observers, over 12-hour shifts in low-volume health facilities and 8-hour shifts in high-volume facilities. A high-volume health facility was one with a daily average of at least 4 deliveries per day or 1 delivery per shift. Only one high-volume health facility was included in the study.

Observation of childbirth was commenced for all consented women when they were admitted into the labour room, whatever the stage of labour, and continued until they were transferred out of the labour room irrespective of the childbirth outcome. Women admitted for elective Caesarean section were excluded from the study.

Women were attended by a number of different attendants, from different professional groups. Observed RMC was evaluated separately for each category of attending health providers, irrespective of the number of attending health providers in each category. Therefore, if three nurses attended to the woman while in labour, only one observational checklist was completed for the nurse professional category. Where a positive and negative observation was obtained for an item on the checklist for each professional category, the negative observation was recorded. This is because we considered RMC as an all-or-nothing requirement so that any negative experience could not be considered as RMC for that item.

### Postpartum interview

Postpartum interviews were successfully conducted on 269 of the 322 women who had been observed. We were not able to interview 53 of the women because they had been referred to another health facility, were discharged home in <12 hours before the interview could be conducted or withdrew participation at that stage. See S1 File for the total number of women observed during childbirth, interviewed postpartum and the previous 12-month average of births at each study health facility. Each observed woman was given a study number, that was tagged to her labour room file, and included in the postpartum data to enable linking of the observation and interview data sets. The interviews were conducted from 12 hours postpartum in the same health facility by two postgraduate students in Public Health. We used a 15-item RMC tool with responses on a 5-point Likert scale of agreement developed by Sheferaw et al. [31]. The sub-categories for the reported RMC 15-item tool are also presented in Table 1. The related sub-categories in the observed and reported RMC tools are placed side-by-side for comparison. Two of the observed RMC sub-categories were not captured in the reported RMC tool.

Additional information obtained at the interview included the women’s socio-demographic and obstetric profiles, as well as the maternal and foetal outcomes of the index pregnancy. The interview questionnaire was translated to the local language (Yoruba) and back-translated into English to aid the interviewers. It was administered in the Yoruba language to the majority of the women. All the instruments were pre-tested at a primary and secondary health facility in Ile-Ife, a neighbouring city to the study location.

### Data management and analysis

Both the observed and postpartum data were captured electronically using the REDCap software [32]. The data was cleaned and analysed using Stata v15. The Stata ‘svy’ commands were used to adjust for clustering and weighting the data against the proportion of deliveries in each facility in the year preceding the study. The observation and interview data sets were merged, but the results presented here are restricted to the 269 women both observed and interviewed.

For the observation tool, each item was scored 1 for a positive response and 0 if negative, for a maximum score of 29. Observations of care by multiple attending health providers must be positive from all attendants for the woman to be said to have received the observed item. For the interview tool, the Likert responses were entered on a numerical scale from 1 to 5.

All RMC scores were converted to a percentage of the maximum possible score to facilitate comparison, for each sub-category and the total scales. The percentage scores for observed RMC were calculated from the number of items in each category. For reported RMC, the mean score was calculated for each category and converted to percentage scores using the formula $\frac{mean-1}{4}\times 100$. This is because the means of the entered Likert responses range from 1 to 5. The calculated percentage scores then range from 0% to 100%.

Although these RMC tools have been widely used, there is no consensus in the literature on the cut-off to be used to define a binary outcome specifying which women received RMC and which didn’t, either by observation or when self-reported. We would presume that for a woman to have received RMC, there should be no deficiencies for any of the items. That is, she must have received 100% of the items on the RMC observational checklist, or agreed or strongly agreed with receiving each item in the interview tool. That would be related to a score ≥75%, using the converted percentage scores. However, none of the women in this study achieved either of those standards. Therefore, the receipt of RMC had to be considered on a continuous scale using the percent scores for both the observational and interview data.

The relationship between the observed and reported RMC percent scores were assessed using Pearson’s correlation statistic. The Bland-Altman plot [33, 34] was used to evaluate the level of agreement between RMC scores obtained by observation of childbirth and from the self-reported postpartum interviews by comparing the difference in their scores on the Y-axis with the mean of the scores on the X-axis. The upper and lower limits of agreement were determined using the 95% confidence limit of the difference in their scores. Factors associated with observed and reported RMC received were assessed using simple and multiple linear regression analysis. Predictors with a p-value of ≤0.2 in bivariate analyses were added to the final multiple regression model simultaneously. However, the level of health facility variable could not be added because it had demonstrated a very high correlation with the birthing facility variable, (0.988). In the regressions, we evaluated the effect of individual facilities on observed and reported RMC scores by contrasting each mean facility score to the overall unweighted mean of the nine health facilities.

### Ethical considerations

Ethical approvals were obtained from the Human Research and Ethics Committees of the University of the Witwatersrand, Johannesburg (M190658), and the Oyo State Ministry of Health (AD/13/479/1386). Permission to visit the health facilities was granted by the Honourable Commissioner for Health, Oyo State Ministry of Health. The identity of the individual health facilities has been anonymised in the reporting of results. The odd-numbered health facilities represent the primary health facilities, while the even-numbered ones represent the secondary health facilities. Consecutive facilities belong to the same LGA in this order- North, North-East, North-West, South-West, South-East.

### Inclusivity in global research

Additional information regarding the ethical, cultural, and scientific considerations specific to inclusivity in global research is included as S2 File.

## Results

### Women’s socio-demographic and obstetric profile

Table 2 presents the unweighted and weighted socio-demographic profiles of the 269 women who were both observed and interviewed. As expected, a higher proportion of the women 170 (63.9%) were aged between 25 to 35 years, with an overall mean age of 28.7 ± 5.7 years. Only 32 (12.0%) of the women had not completed secondary or post-secondary education. Almost all the women (92.3%) were employed at the time of the study. The facility characteristics are also shown in Table 2. A higher proportion of the women 189 (70.2%) were recruited from secondary health facilities. The highest proportion of women were from Ibadan North LGA (47.1%) and facility 2, 112 (41.7%).

**Table 2:** Women’s socio-demographic profile and delivery site characteristics (n = 269).

	Unweighted		Weighted	
**Socio-demographic variables**	**Freq.**	**%**	**Freq.**	**%**
**Age in years**				
Youths (18–24 years)	62	23.3	60	22.4
Adults (25–35 years)	167	62.8	170	63.9
Adults (>35 years)	37	13.9	36	13.7
Mean ± SD; median (IQR)	28.6 ±5.8; 28 (25, 33)		28.7 ± 5.7; 28 (25, 33)	
**Highest level of education completed**				
None/ Primary education	34	12.6	32	12.0
Secondary	120	44.6	118	43.9
Post-secondary education	115	42.8	119	44.1
Secondary or post-secondary education				
**Employment status**				
Employed	249	92.6	248	92.3
Unemployed	20	7.4	21	7.7
**Personal Income (US $)**				
≤$1.90/day/month (World bank poverty level)	191	74.6	189	73.6
>$1.90/day/month	65	25.4	68	26.4
Monthly income US $. Mean ± SD; median(IQR)	50.1 ± 62.3; 39.5 (15.8, 61.8)		50.9 ± 63.6; 39.5 (15.8, 61.8)	
**Ethnicity**				
Yoruba	252	93.7	251	93.5
Ibo	17	6.3	18	6.5
**Delivery facility type**				
Primary health facility	87	32.3	80	29.8
Secondary health facility	182	67.7	189	70.2
**LGA**				
Ibadan North	139	51.7	127	47.1
Ibadan North East	21	7.8	24	8.8
Ibadan North West	37	13.7	38	14.2
Ibadan South East	33	12.3	30	11.2
Ibadan South West	39	14.5	50	18.7
**Delivery health facility**				
Facility 1	16	6.0	15	5.4
Facility 2	123	45.7	112	41.7
Facility 3	11	4.0	11	4.1
Facility 4	10	3.7	13	4.8
Facility 5	21	7.8	19	7.1
Facility 6	16	6.0	19	7.1
Facility 7	6	2.3	5	2.0
Facility 8	33	12.3	45	16.6
Facility 9	33	12.3	30	11.2

₦380 = 1US $

**Fig 1:**
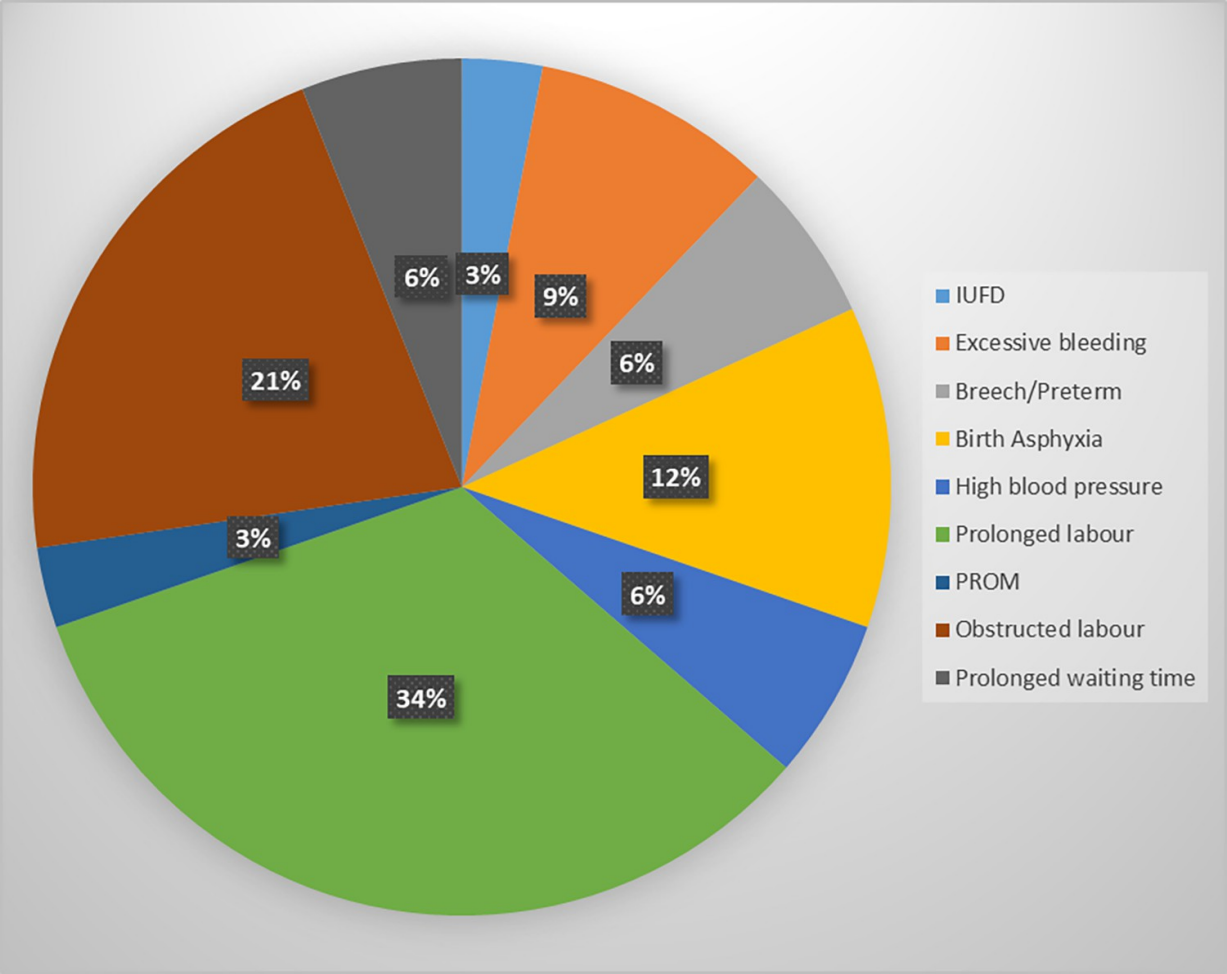
Reported birth complications.

Table 3 shows the weighted obstetric and labour history of the study participants. A higher proportion of the women 157 (58.7%) were multiparous, and about 50% of them had previously delivered in the same study health facility. However, one-fifth of them 33 (20.9%) did not deliver in a health facility for their previous delivery. Nonetheless, 204 (92.5%) of the women had booked at a health facility for their index pregnancy. Thirteen (4.9%) of the women were family relations of the health providers, while 237 (88.1%) were not familiar with them at all. A higher proportion of the women presented during the daytime in labour 164 (60.9%) and at term 217 (81.3%), but 55 (20.4%) presented late in labour with >8cm dilatation. Uncomplicated childbirth was the more common outcome for 230 (86.8%), and 257 (97.2%) had a live birth (97.2%). Fig 1 shows the complications experienced as reported by the women during the postpartum interview. They either used the exact terms the health providers had told them or described them using their own words. The most common complication experienced was prolonged labour (34.0%), followed by obstructed labour (21.0%).

**Table 3:** Women’s characteristics in labour and obstetric history reported at postpartum (n = 269).

Variables	Freq. (n)	(%)
**Obstetric history**		
**Parity**		
Primiparous	111	41.3
Multiparous	157	58.7
Total pregnancies ever had (multips) (Mean ± SD)	2.2 ± 1.2	
Total number of previous live births ever had (multips) (Mean ± SD)	1.8 ± 1.0	
**Had previously delivered in the study health facility (multips)**		
Yes	79	50.5
No	78	49.5
Number of times delivered in the study health facility (Mean ± SD)	1.6 ± 0.5	
**Place of delivery for last births (multips)**		
Health facility	124	79.1
Non-health facility	33	20.9
**Current pregnancy**		
**Booking status in the current pregnancy**		
Booked	204	92.5
Unbooked	16	7.5
**Familiarity with providers at the delivery site**		
Not familiar	237	88.1
Familiar: non-family relationship	19	7.0
Familiar: family relationship	13	4.9
**Time of presentation in labour**		
7:00am.– 18:59pm (Daytime)	164	60.9
19:00pm– 6:59am (Night/midnight)	105	39.1
**Gestational age at labour (weeks)**		
Pre-term (<37 weeks)	36	13.6
Term (37–40 weeks)	217	81.3
After term/ late term (>40 weeks)	14	5.1
**Cervical dilatation at presentation**		
<8cm dilated	214	79.6
8cm -10cm dilated	55	20.4
**No of the attending provider types**		
1 provider category	215	80.0
2 provider categories	54	20.0
**Combination of attending providers during childbirth**		
Nurse only	149	55.3
Community Health Worker (CHW) only	62	23.3
Health Auxiliary (HA) only	4	1.4
Doctor/ Nurse	44	16.2
Nurse/ CHW	4	1.4
CHW/ Health Auxiliary	6	2.4
**Maternal outcome of the current delivery process**		
Uncomplicated delivery	230	86.8
Complicated delivery	35	13.2
**Foetal outcome of the current childbirth process**		
Live birth	257	97.2
Still birth	7	2.8

In terms of the possible impact of their labour experience on future obstetric choices, the majority of the women 222 (83.8%) stated that they would return to the same health facility for possible future delivery. Also, 243 (92.7%) of them would refer other women to the same health facility for delivery.

### Observed RMC received

Table 4 shows the number and proportion of women who received each of the 29 items in the observational checklist. The mean percent scores for each of the observed RMC sub-categories are shown below the items for each sub-category, and the overall percentage for the entire scale is given at the bottom of the table.

**Table 4:** Observed RMC items and categories received by women during childbirth (n = 269).

	Observed RMC received during labour and childbirth	Freq.	%
	**A. Women’s right to information and informed consent (Autonomy)**		
1	Welcomed/ greeted on presentation in labour	38	14.0
2	Provider(s) introduced themselves to the woman (and her companion)	1	0.3
3	Companions are encouraged to remain with the woman for as long as possible	22	8.2
4	Encouraged the woman to ask questions	1	0.3
5	If asked, responded with promptness, politeness, and truthfulness	116	43.1
6	Explained what was being done and what to expect in labour and birth	29	10.8
7	Gives periodic updates on status and progress of labour	87	32.5
8	Allowed women to move about during labour	13	4.9
9	Allowed to assume the birth position of choice	5	1.9
10	Provided with information before conducting procedures	89	33.2
11	Consent or permissions obtained from women before procedures	11	4.0
	**Mean ± SD of information & consent received (max:100%)**	**14.0 ± 12.3**	
	**B. Women protected from physical harm and ill-treatment**		
1	Was never slapped, hit (nor attempted) during labour	194	72.0
2	Was never physically restrained	246	91.5
3	Was touched and caring demonstrated in a culturally appropriate way	67	25.0
4	Was never separated from her baby except medically necessary	224	83.3
5	Was not denied food or fluid in labour unless medically necessitated	217	80.7
6	Was given pain relief and provided comfort as necessary	22	8.2
	**Mean ± SD of no physical harm received (max:100%)**	**60.1± 20.5**	
	**C. Privacy and confidentiality protected**		
1	Her file was kept in a locked cabinet and not displayed carelessly	7	2.7
2	Was protected with curtains during all examinations	4	1.4
3	Appropriate drapes or coverings were used to protect her privacy	74	27.5
4	Her personal and medical details were never discussed openly	219	81.2
	**Mean ± SD of privacy &confidentiality received (max:100%)**	**28.2 ± 16.1**	
	**D. Woman is treated with dignity and respect**		
1	Woman and/or companion was/were spoken to politely	175	65.0
2	Woman and/or companions allowed to observe their cultural and religious practices	86	31.9
3	Woman and/or companions were never insulted, intimidated, threatened, or coerced	169	63.0
	**Mean ± SD of dignity & respect received (max:100%)**	**53.3 ± 30.5**	
	**E. Receives equitable care free from discrimination sub-category**		
1	Spoken to in a language and at a language level that she understands	259	96.1
2	Not disrespected based on any specific personal attribute of hers	227	84.4
	**Mean ± SD of non-discriminatory care received (max:100%)**	**90.3 ± 23.9**	
	**F. Woman never left without care (Non-neglect of care)**		
1	Was encouraged to call providers if needed	28	10.4
2	Provider(s) come quickly when the woman calls	171	63.5
3	Was never left unattended to	177	65.0
	**Mean ± SD of non-abandonment of care received (max:100%)**	**46.6 ± 33.7**	
	**Received 50% or more of the 29 items in the MCHIP RMC checklist**	**41**	**15.2**
	**Received 100% of the 29 items in the MCHIP RMC checklist**	**0**	**0.0**
	**Mean ± SD Observed RMC percent scores received (max:100%)**	**38.2 ± 12.1**	

The RMC sub-category with the lowest mean percentage score was the *information and informed consent* sub-category, where the women were observed to receive an average of only 14.0% of the 11 RMC items (Table 4). Although 89 (33.2%) were given information about procedures, only 11 (4.0%) were always asked for consent before the procedures were undertaken. In only one case (0.3%) was the provider observed to have introduced herself or encouraged the woman to ask questions, and only 5 (1.9%) were allowed to assume the birth position of choice.

The next weakest RMC sub-category was *privacy and confidentiality* with a mean percent score of 28.2%. Only 4 (1.4%) of the women were properly screened during examinations, though 219 (89.2%) did not have their personal and medical details discussed openly.

The average score for the *being protected from physical harm* sub-category was 60.1% across the 6 items where 246 (92.5%) were never physically restrained and 224 (83.3%) were not separated from their babies unnecessarily. However, 75 (28.0%; 95%CI: 23.0%-33.0%) of women were observed to be hit during labour, and only 22 (8.2%; 95%CI: 4.0%-18.0%) received pain relief.

*Non-discriminatory care* was the highest sub-category of RMC received, with a mean of 90.3% for the two items. Here, 259 (96.1%) of women were spoken to in a language they could understand and 227 (84.4%) were not disrespected based on their personal attributes.

Overall, the women were observed to receive an average of 38.2% of the 29 RMC items on the scale. Only 41 (15.2%) of the women received 50% or more of the 29 items in the observational checklist, while no woman received more than 60% of the RMC items (Table 4).

### Reported RMC received

The results for RMC reported in the post-partum interview are presented in Table 5. The table shows the number and proportion of women who agreed or strongly agreed with each of the 15 items on the checklist together with the mean score for each item, calculated as a percentage of the maximum possible score. The mean percent scores for each of the reported RMC sub-categories are shown below the items, and the overall percentage for the entire scale is given at the bottom of the table.

**Table 5:** Reported perceptions of RMC received during childbirth from post-partum interview (n = 269).

S/n	Reported perceptions of RMC received	Freq. (%) agreed/ strongly agreed	Percentage of the maximum score Mean ± SD
**A**	**Friendly care**		
1	Approached kindly by health care providers (nice and ready to help)	251 (93.4)	78.9 ± 19.1
2	Healthcare workers were friendly (pleasant) in their treatment	253 (94.1)	78.1 ± 18.4
3	Healthcare providers encouraged me positively about pain and its relief.	251 (93.4)	78.4 ± 17.6
4	Healthcare providers were concerned and empathetic	251 (93.4)	77.7 ± 19.4
5	Was respected as an individual (treated like a human being)	246 (91.4)	76.0 ± 21.0
6	Was communicated with the woman in an understanding language	264 (98.3)	81.0 ± 14.5
7	Was addressed by name (not say things like, "eh that woman")	216 (80.4)	67.1 ± 31.2
	**Mean ± SD percent score of friendly care sub-category**	**76.7 ± 14.9**	
**B**	**Abuse-free care**		
1	Health needs were responded to appropriately and professionally	251 (93.4)	76.9 ± 20.9
2	Was never hit (pinched, slapped, punched, beat) during labour	234 (87.0)	78.7 ± 29.5
3	Was never yelled at for not obeying provider instructions	224 (83.1)	74.7 ± 32.2
	**Mean ± SD percent score of abuse free care sub-category**	**76.8 ± 20.9**	
**C**	**Timely care**		
1	Was given prompt service and never neglected during labour	251 (93.2)	81.9 ± 26.0
2	Was free to practice safe cultural & religious traditions during labour	237 (88.1)	79.9 ± 27.7
3	Problems like many patients never delayed providers’ service to clients	257 (95.6)	84.6 ± 22.8
	**Mean ± SD percent score of timely care sub-category**	**82.1 ± 17.2**	
**D**	**Non-discriminatory care**		
1	Was never mistreated because of clients’ personal characteristics	262 (97.4)	89.5 ± 17.6
2	Clients’ companions were never offended due to personal characteristics	251 (93.3)	86.1 ± 21.9
	**Mean ± SD percent score of non-discriminatory care**	**87.3 ± 17.0**	
	**Agreed to have received 50% or more of the 15-item RMC scale**	**264**	**98.0**
	**Agreed to have received 100% of the 15-item RMC scale**	**5**	**2.0**
	**Mean ± SD score of RMC reported**	**79.2 ± 11. 7**	

A high proportion of women agreed that they had received each of the 15 RMC items–the figure was above 90% for most items, and none was below 80% (Table 5). The mean scores for each item, expressed as a percentage of the maximum possible score, were slightly lower but most were above 75%. The lowest scoring RMC item was being addressed by name where 80.4% (95%CI: 67.3%-89.1%) of women agreed to it, for a mean percent score of 67.1%. Only 13% of women (95%CI: 9.2%-18.0%) agreed that they had been hit during labour.

The mean percent scores for each sub-category were correspondingly high, ranging from 76.7% for *friendly care* and 76.8% for *abuse-free care* to 82.1% for *timely care* and 87.3% for *non-discriminatory care*.

Overall, the mean percent score for all 15 items was 79.2%. Although almost all of the women (98%) agreed that they had received at least 50% of the reported RMC items, only 5 (2.0%) agreed to having received all of the items.

### Relationship between reported and observed RMC scores

**Fig 2:**
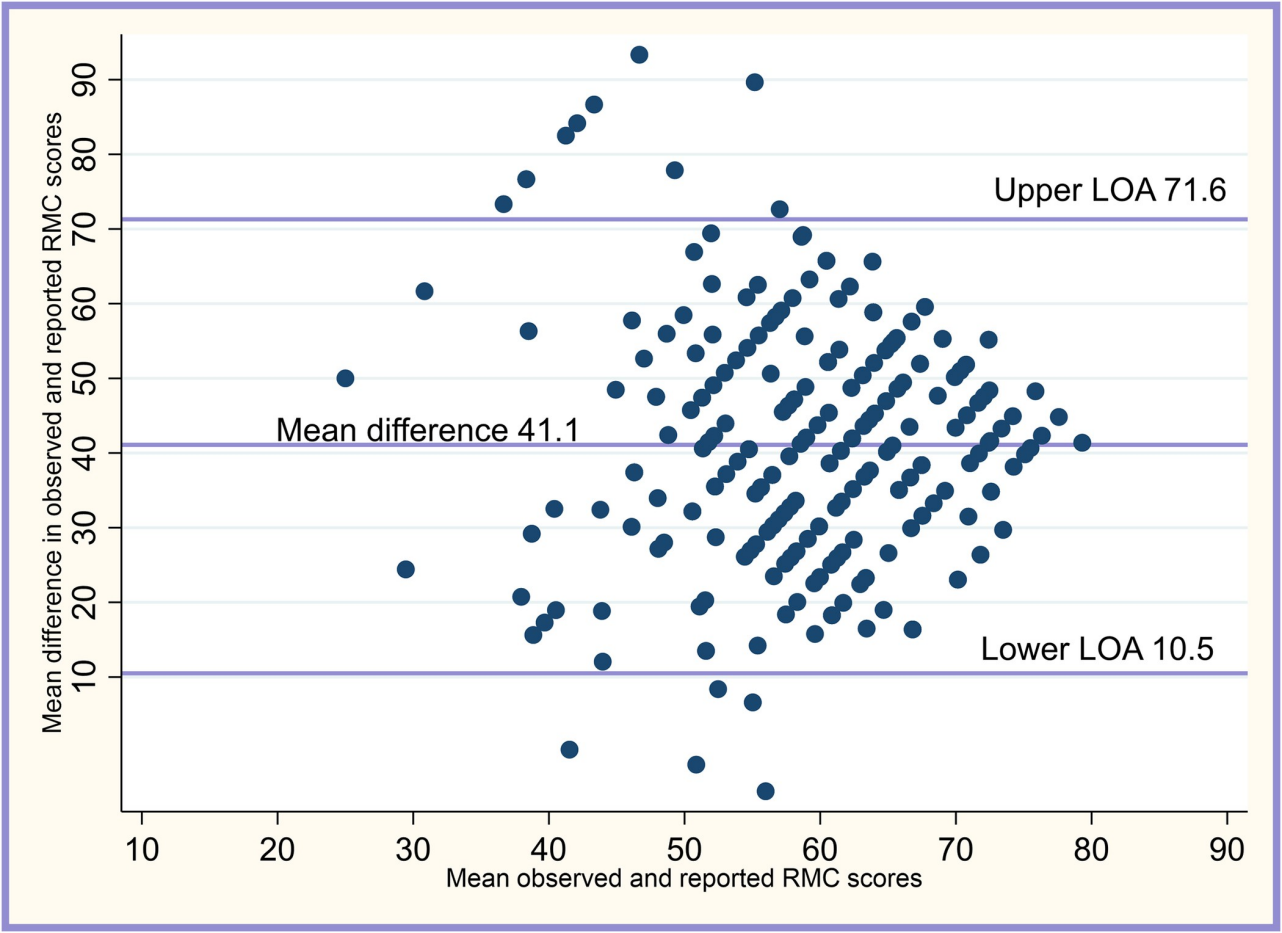
Bland-Altman plot on levels of agreement between observed and reported RMC received.

Fig 2 is the Bland Altman plot comparing the reported and observed RMC scores. This shows very weak agreement between the two measures. On average, the difference between the reported and observed scores was 41.1 percentage points. Not surprisingly, the mean difference was statistically significantly different to zero (one-sample t-test = 43.2, p-value <0.001). However, there was a weak but significant positive correlation between the reported and observed RMC scores (rho = 0.164, 95%CI: 0.045–0.278, p = 0.007).

### Factors associated with observed RMC received

Table 6 presents the simple and multiple regression analysis of the factors associated with the observed RMC score. The available covariates included the women’s personal characteristics, obstetric history, and facility attributes. RMC scores were lower for both the younger and older age groups compared to the middle 25–35 years’ age group though this was not statistically significant. Women’s employment and educational status were the two socio-demographic characteristics significantly associated with the observed RMC scores received in the multiple regression analysis. The score for employed women was 4.3 percentage points higher than for those who were unemployed (p = 0.012). The RMC scores for women with post-secondary education were 6.4 percentage points higher than those with primary school education or less (p = 0.014). Being related to the health providers at the delivery facility earned the women higher observed RMC scores that tended towards being significant (p = 0.056). Women who were attended to by only one category of health provider had significantly higher observed RMC scores than those attended to by two categories of health providers, (p = 0.014). Women who delivered in Facilities 2,4,5 and 6 received significantly higher observed RMC scores when contrasted to the overall unweighted mean RMC scores for the nine health facilities, with Facility 4 being the highest. However, women delivering in facilities 1,7, and 8 received significantly lower observed RMC scores than average, with Facility 1 being the lowest. Overall, the health facility where the women delivered was a significant predictor of RMC scores received, (F = 486.6; DF = 7; p = 0.035).

**Table 6:** Factors associated with observed RMC scores during childbirth.

Covariates	Bivariate Analysis			Multiple Regression Analysis		
	Crude Coeff.	95%CI	p-value	Adjusted Coeff.	95%CI	p-value
**Age in years**						
Youths (18–24 years)	-3.0	-6.3–0.3	0.070	-1.0	-5.1–3.0	0.559
Adults (25–35 years)	Ref	-	-	Ref	-	-
Adults (>35 years)	-1.8	-10.7–7.0	0.638	-2.9	-7.5–1.7	0.174
**Education completed**						
None/Primary education	Ref	-	-	-	-	-
Secondary education	5.2	1.5–8.7	**0.012**	3.3	-1.3–7.8	0.135
Post-secondary education	7.0	2.7–11.4	**0.007**	6.4	1.7–11.1	**0.014**
**Employment status**						
Employed	5.6	-1.530–12.8	0.105	4.3	1.3–7.4	**0.012**
Not employed	Ref	-	-	Ref	-	-
**Monthly Income (usd) per 1000**	15.3	4.0–26.4	**0.014**	-6.6	-19.8–6.5	0.271
**Ethnicity**						
Yoruba	-1.8	-8.1–4.4	0.510			
Ibo	Ref	-	-			
*Delivery health facility						
Facility 1	-8.6	-15.7–-1.4	**0.019**	-9.5	-10.2–-8.7	**<0.001**
Facility 2	2.7	0.04–5.3	0.047	1.8	0.8–2.7	**0.004**
Facility 3	-3.7	-8.8–1.4	0.155	-0.03	-3.1–3.0	0.980
Facility 4	5.9	1.9–9.8	**0.004**	10.1	6.6–13.5	**0.0002**
Facility 5	2.0	-1.4–5.4	0.246	1.1	0.3–2.0	**0.019**
Facility 6	6.3	2.8–9.8	**0.001**	5.8	4.6–7.0	**<0.001**
Facility 7	-2.2	-8.2–3.8	0.473	-4.6	-6.2–-3.0	**0.0002**
Facility 8	-2.1	-5.9–1.6	0.262	-2.8	-4.2–-1.3	**0.003**
Facility 9	-0.2	-3.8–3.2	0.892	-1.8	-3.7–0.1	0.059
**Facility type**						
Primary	Ref	-	-			
Secondary	3.9	-1.5–9.4	0.130			
**Attending provider**						
1 provider category	10.4	-1.2–22.0	0.071	12.5	3.4–21.6	**0.014**
2 provider categories	Ref	-	-	Ref	-	-
**Provider familiarity**						
Not familiar	Ref	-	-	Ref	-	-
Familiar (non-family)	3.5	-1.3–8.4	0.131	1.9	-0.8–4.5	0.138
Familiar (as family)	4.6	-2.1–11.4	0.147	5.0	0.2–10.2	**0.056**
**Parity**						
Primipara	Ref	-	-			
Multipara	1.5	-1.4–4.4	0.251			
**Booking status**						
Booked	2.0	-10.4–14.4	0.713			
Unbooked	Ref	-	-			
**Presenting time-labour**						
7:00am–18:59pm (DT)	Ref	-	-			
19:00pm– 6:59am (NT)	-0.3	-5.7–5.1	0.906			
**Constant**				10.6	3.0–18.2	**0.013**
				*n = 253; R*^*2*^ *= 0*.*235;* ***p<0*.*001***		

Significant p values in bold. DT- daytime, NT- Night time. *These results are shown as contrasts against the mean

The results for reported RMC are provided in S2 File. Higher RMC scores were reported by women of Yoruba ethnicity (p = 0.045), and those who were familiar with the provider(s) at the birthing facility (p = 0.045). Women who delivered in Facilities 2,4,6, 8 and 9 reported significantly higher RMC scores, with Facility 2 being the highest. However, women who delivered in Facilities 1,3,5 and 7 reported significantly lower RMC scores, with Facility 1 being the lowest. Overall, the health facility where the women delivered was a significant predictor of reported RMC scores received, (F = 7151.3; DF = 7; p<0.001).

## Discussion

This study measured the level of RMC received during childbirth using both observation and postpartum interviews and evaluated if any differences in the observed RMC could be attributed to characteristics of the women or delivery facility. The level of RMC received by observation of childbirth was low, with an average score of only 38.2%. No woman was observed to have received 100% of the RMC checklist. However, the interview tool produced much higher levels of reported RMC–the women scored 79.2% of the maximum possible for the self-reported RMC tool. There was very little agreement between the two methods of measurement (observation and self-reported postpartum interview) even though the scores were significantly positively correlated. Employed women, and those who had post-secondary education were observed to have received significantly higher levels of RMC. Familiar relationship with the attending health providers and the delivery facility were also significant predictors of observed and self-reported RMC received.

### Defining the receipt of RMC-a methodological challenge

Although the observation and self-reported tools have been widely used to measure RMC, there is no consensus on the benchmark to be used to define who received RMC or not, for either tool [20, 35]. Hence, we did not characterise which women received RMC based on a defined cut-off. Different studies in the literature have used varying standards to determine who received RMC, although the rationales for doing so are not always clearly stated. Some studies only describe the proportion who received each item without a composite analysis of RMC across the items [36, 37]. Others reported either a few or the total number of items in the Maternal and Child Health Integrated Program (MCHIP) RMC observational checklist to assess the level of RMC received [38, 39]. One study observed women during childbirth using all the items on the MCHIP tool but defined women who received RMC as those who received at least the mean score for each of the 7 sub-categories [40]. For the 15-item RMC scale, most studies dichotomised the 5-Likert scale into agreed and disagreed responses to assess the items of RMC received [17, 41]. Some transformed the total scores to 100 [18], and others also defined who received RMC from the mean percent scores [42]. One study determined the self-reported RMC mean scores received and categorised them into very low and very high [16].

This raises questions about the conceptualisation and measurement of RMC that have received inadequate attention in the current RMC literature. We started with the premise that the receipt of RMC is an absolute rather than a relative construct. Therefore, rather than comparing to some arbitrary cut-off, RMC should properly be considered as an all or none concept. So that all of the defined components of RMC have to be achieved before women could be said to have received RMC. For example, we would not consider a woman to have received RMC if she received most of the individual RMC items or sub-categories, but was physically beaten during labour. However, none of the women observed in our study came close to achieving that standard (receiving all items assessed), so we resorted to measuring the level of RMC received on a relative scale. That metric then reflects progress toward RMC rather than actually achieving it, which may be necessary given the current levels of RMC in most LMICs.

Another issue in measuring RMC is the appropriate weighting to be used for different RMC items or sub-categories, and who should determine them. We used the equal weighting of items usually assumed for the existing observation and interview tools but were concerned that some RMC dimensions should perhaps be afforded more importance in evaluating the receipt of RMC (physical harm, for example), and that the relative importance of different RMC sub-categories did not necessarily match the number of items included in the tools for each sub-category. These considerations require more debate, investigation and guidance in the RMC measurement literature.

### Comparison of observed and reported RMC scores

Another methodological difficulty in measuring RMC is that significantly different results are obtained depending on which method is used. There were much higher mean scores for the overall RMC that women reported to have received (79.2% of all items) compared to what was independently observed (38.2%). This difference was also noted when comparing similar sub-categories in both the observed and reported RMC tools, as matched in Table 1. For example, the average score for the no physical harm sub-category was 60.1% from observation, but 76.8% for abuse-free care when reported by women themselves. Indeed, 28% were observed to have been hit or slapped during childbirth, however, only 13% reported this at postpartum.

The items are not exactly the same in the two tools, the metrics also differ as the observation tool had a dichotomous response while the reported interview was measured on a Likert scale. For reported RMC, there was also the possibility of a social desirability bias, or fear of repercussions if they were critical about their childbirth experience. The discrepancy between self-reported and observed RMC may also suggest some normalisation of abuse among the women in our study, since they report high levels of satisfaction with the care they receive but disregard the persistent mistreatment and denial of basic rights during childbirth.

This disparity in the observed and self-reported prevalence of disrespectful care during childbirth was also found in an RMC-promoting intervention study in Tanzania. In that study, the prevalence of disrespectful care by observation and self-report respectively was 69.8% versus 9.9% at baseline and 32.9% versus 7.6% after the intervention [43]. They concluded that both the women and health providers have internalised and normalised women’s mistreatment during childbirth.

Several studies had called for more observational studies to assess the receipt of RMC during childbirth [8, 40, 43, 44]. However, these methods have their limitations, including a possible Hawthorne effect and high cost [40, 43–45]. Observational studies may be preferred because the events are being measured as they occur and the measurements are more objective, as long as the Hawthorne effect can be controlled. Social desirability bias may affect the validity of self-reported postpartum interviews when conducted both within and outside health facilities [20], and recall biases may interfere when interviews are conducted later in women’s homes. Irrespective of the interview location, measurement of RMC may be compromised by the halo effect following a successful birth or because women don’t clearly understand their rights, leading to under-reporting of mistreatment. Whichever methods are used, the various limitations must always be considered when interpreting the results of RMC evaluations [44].

Our study showed a mean difference of 41.1% in the observed and reported RMC scores received using the 29-item MCHIP observational checklist and the 15-item RMC scale for a postpartum interview respectively. This shows that these two methodologies (observation and postpartum interviews) poorly agree, and may not be used as proxies for each other. This is consistent with other findings in the literature which suggest that the two methods- observational and postpartum interviews are not comparable [43]. They are two imperfect methodologies measuring different things.

### Levels of the observed and reported RMC in the study site

There was a very poor performance with the observed RMC scores received in our study, with a mean score of only 38.2% for the 29 items in the observational checklist. A similar RMC observational study conducted in the Benishangul Gumuz region of Ethiopia using all the items on the MCHIP checklist reported that a very low proportion of women (12.6%) received RMC [46]. Their assessment of RMC was based on receiving the mean score or higher for each of the 7-sub-categories assessed, which is quite different to the method of analysis we have presented. However, 41 (15.2%) of women in our study would have been considered to have received RMC using their metric. The low proportion of women observed to have received RMC in these studies is worrisome and reflects the level of mistreatment women receive during childbirth. This is corroborated by the 28% of the women we studied who were hit or slapped. There is a need for more collaborative global efforts to combat women’s mistreatment and deliver RMC to them, especially during childbirth.

The mean self-reported RMC score received among the women in our study (79.0%) was higher than the moderate degree (56.0%) reported by postpartum women at a tertiary health facility in Egypt [16]. However, a higher proportion of the women studied in Egypt were in the middle socioeconomic class while 75% of ours earned <$1.9/day.

The best RMC sub-category, received by most women in our study, for both observed and reported RMC, was equitable/non-discriminatory care. Almost all the 404 women observed, (97%) during childbirth at public hospitals in the Benishangul Gumuz region in Ethiopia also received non-discriminatory care [46]. Privacy during childbirth was not guaranteed when delivering at our study health facilities. This is corroborated by a previous observational study in four countries that found that privacy measures such as the use of curtains to screen for procedures during childbirth were seldom available in Nigeria [21]. In our study, few women (33.2%) were informed adequately before procedures were conducted and only 4% were observed to be properly asked for their consent. This was also similar to the findings in the Ethiopian study earlier described as only 17.5% of the women had their consent obtained before procedures during the observation of their birth [46].

Not obtaining the women’s consent denies them the right to autonomy and decision-making, while not protecting their privacy shows a lack of respect for their person. Gaining women’s consent is a legal and ethical issue [47]. Valid consent is only obtained when women have been adequately informed of the risks and benefits of procedures during childbirth [47]. Unfortunately, there has been the challenge of how much information to give [47, 48], and the real or perceived safety concerns if consent is not granted during childbirth, for example for episiotomy. The health providers have a responsibility to protect the unborn child without abusing women’s rights. This calls for more discussion with health providers about ensuring women’s rights to autonomy while yet preserving the safety of the unborn child.

### Factors associated with observed RMC received

Being gainfully employed was significantly associated with observed RMC received in our study. However, women’s occupation was not significantly associated with receiving RMC in the Benishangul Gumuz observational study of childbirth [46]. Women’s level of education was significantly directly proportional to the level of observed RMC scores received in our study. Lack of education was also one of the primary determinants of mistreatment received by women during childbirth according to the WHO-supported study conducted across four countries [21] Higher levels of education and being gainfully employed would expectedly increase women’s ability to demand their rights during childbirth and procure essential commodities for their birth [49]. In health facilities where essential birthing requirements are not freely provided to women, or where there are no financial protection schemes for women, non-procurement of these delivery materials has contributed to women’s mistreatment during childbirth [30]. Nonetheless, every woman should receive RMC irrespective of their personal attributes. Familiarity with the health providers increased the women’s chances of receiving and reporting having received RMC during childbirth in our study. This is not surprising but undermines the delivery of equitable care.

There were significant differences in the amount of RMC women received across the study facilities. Delivering at general hospitals rather than primary health facilities was also associated with the receipt of more RMC in the Benishangul Gumuz study [46], though we could not determine the difference in RMC scores between secondary and primary health facilities in our study. The significant overall facility effect identified in our study implies that quality and respectful childbirth care is not standardised across the public health facilities in the study setting. This may be addressed with the use of RMC guidelines by trained health providers. The contextual differences in individual health facilities contributing to this may also need to be investigated and addressed.

## Strengths and limitations of the study

Our study used two methodologies with separate instruments for the same women revealing the strengths and weaknesses of one over the other. We captured practices from both the primary and secondary health facilities, presenting a diversity of contexts. The use of registered nurses as observers with an understanding of the clinical processes was a strength. We however provided extensive training to ensure that their perceptions of what constitutes mistreatment during childbirth would not influence their observations.

There are some study limitations. The possibility of a Hawthorne effect is a concern in observational studies. This was mitigated by staying for a relatively long period in each facility (1 month) so that providers got used to the presence of the observers. We attempted to reduce observer bias by ensuring that the selected observers had never worked nor trained at the study health facilities, as this may influence their judgements. Observers were also rotated between the two health facilities studied each month. We observed very low levels of RMC, suggesting that provider behaviour was not significantly influenced by being observed. We used the available and commonly-used standardised RMC tools for observation and interviews but these do have some limitations. The reported RMC instrument did not capture critical components in the global definitions of RMC which include respect for women’s autonomy, and privacy with confidentiality. This hampered the full comparison of both tools. The reliability of most subscales was good, while for a few was poor. However, the reliability of the overall observed and reported RMC scales was adequate (Table 1). A number of the women observed could not be interviewed because they were referred out and could not be traced at the referral facility. Lastly, the study excluded public tertiary health facilities and private hospitals which may limit the generalisation of study findings to these categories of health facilities.

## Conclusion

Independent observers found low levels of RMC during childbirth for the women in our study from Ibadan, Nigeria. However, the women themselves reported high levels of RMC. The study findings highlight several important methodological issues for the measurement of RMC in LMICs, including the comparability of different methods, the cut-offs to be used in defining the receipt of RMC, and the appropriate weighting of tool items. Self-reported RMC measures are widely used in the literature but may over-report the level of RMC received because of social-desirability bias and the normalisation of abuse and mistreatment in many LMIC settings.

We identified problems with certain aspects of delivering RMC during childbirth in the Nigerian study facilities which include providing adequate information, always obtaining consent before procedures, privacy during examinations, allowing women to choose alternative birth positions and normalisation of abuse. Non-discriminatory care was observed and reported by the women. However, women who are employed, better educated and familiar with the health providers enjoyed more respectful care during childbirth. There were significant individual facility effects on the extent of RMC received during childbirth. Thus, the role of standardisation of practice and the facility contextual factors on the universality of RMC implementation may require further investigation.

## Supporting information

S1 File Breakdown of observations, postpartum interviews, and previous 12-month average facility delivery per study facility. (DOCX)

S2 File PLOS questionnaire on inclusivity in global research. (DOCX)

S3 File Factors associated with reported RMC received at birth. (DOCX)
