# Supplementary material for: Directly observed and reported respectful maternity care received during childbirth in public health facilities, Ibadan Metropolis, Nigeria
Source: PLoS One. 2022 Oct 21;17(10):e0276346. doi: 10.1371/journal.pone.0276346 (PMC9586397; doi:10.1371/journal.pone.0276346)
Supplement: S1 File — (DOCX) [file pone.0276346.s001.docx]

**Supplement 1**

Table: Breakdown of unweighted women observed and the weighted and unweighted women eventually interviewed amongst the women observed by facility

| **Facilities** | ***Average Births in the previous 12-months** | **Observed women within study period** | **Postpartum interviewed women**  **within study period** | |
| --- | --- | --- | --- | --- |
|  | **Unweighted** | **Unweighted** | **Unweighted** | **Weighted** |
| Facility 1 | 16 (5.4%) | 17 (5.3%) | 16 (6.0%) | 15 (5.4%) |
| Facility 2 | 123 (41.7%) | 158 (49.1%) | 123 (45.7%) | 112 (41.7%) |
| Facility 3 | 12 (4.1%) | 11 (3.4%) | 11 (4.0%) | 11 (4.1%) |
| Facility 4 | 14 (4.7%) | 10 (3.1%) | 10 (3.7%) | 13 (4.8%) |
| Facility 5 | 21 (7.1%) | 22 (6.8%) | 21 (7.8%) | 19 (7.1%) |
| Facility 6 | 21 (7.1%) | 23 (7.2%) | 16 (6.0%) | 19 (7.1%) |
| Facility 7 | 6 (2.0%) | 6 (1.8%) | 6 (2.3%) | 5 (2.0%) |
| Facility 8 | 49 (16.8%) | 40 (12.4%) | 33 (12.3%) | 45 (16.6%) |
| Facility 9 | 33 (11.2%) | 35 (10.9%) | 33 (12.3%) | 30 (11.2%) |
| Total | 295 (100.0%) | 322 (100.0%) | 269 (100.0%) | 269 (100.0%) |

*** The immediate 12-month average of the births before the commencement of the observations in each study facility**
