# Supplementary material for: Directly observed and reported respectful maternity care received during childbirth in public health facilities, Ibadan Metropolis, Nigeria
Source: PLoS One. 2022 Oct 21;17(10):e0276346. doi: 10.1371/journal.pone.0276346 (PMC9586397; doi:10.1371/journal.pone.0276346)
Supplement: S2 File — (DOCX) [file pone.0276346.s002.docx]

Inclusivity in global research

PLOS’ policy on inclusivity in global research aims to improve transparency in the reporting of research performed outside of researchers’ own country or community and ensures that PLOS publications reporting global research adhere to high standards for research ethics and authorship. Authors of relevant research articles may be asked to complete the questionnaire below, which outlines ethical, cultural, and scientific considerations specific to inclusivity in global research. This questionnaire may be requested when researchers have travelled to a different country to conduct research, if research uses samples collected in another country, research with Indigenous populations or their lands, or if research is on cultural artefacts. Researchers travelling to another country solely to use laboratory equipment will not normally be required to complete the questionnaire. However, the questionnaire can be requested at the journal’s discretion for any submission – if you have been requested to complete this questionnaire by the PLOS journal you submitted to, please do so.

Please complete the questionnaire below and include this as a Supporting Information file with your manuscript. Note that if your paper is accepted for publication, this checklist will be published with your article in the supporting information files. Please ensure that you reference the checklist in the main body of your manuscript. We suggest adding a subsection ‘Inclusivity in global research’ to your Methods section and adding the following sentence: “Additional information regarding the ethical, cultural, and scientific considerations specific to inclusivity in global research is included in the Supporting Information (SX Checklist)”

The questions have been designed to be applicable to a wide range of study types, and there are subsections for both human subjects’ research and non-human subjects research. If any of the questions are not relevant to your research, please mark them as “N/A” as appropriate.

**Ethical considerations, permits and authorship**

*This section is applicable to all research types.*

Provide details as to who granted permission and/or consent for the study to take place in the Methods section of your manuscript. This should include the names of **all** ethics boards, governmental organizations, community leaders or other bodies that provided approval for the study. If individuals provided approval refer to these people by their role or title but do not list their name(s).

Ethical approvals were obtained from the Human Research and Ethics Committees of the University of the Witwatersrand, Johannesburg (M190658), and the Oyo State Ministry of Health (AD/13/479/1386). Permission to visit the health facilities was granted by the Honorable Commissioner for Health, Oyo State Ministry of Health. The first author who was directly involved with the data collection for this study did not have to travel from any country to the study site. She is a resident of the country where the study was conducted and lived in a neighbouring state about 1 hour to the study location.

Reported on page number: Page 11

If there were any deviations from the study protocol after approval was obtained please provide details of these changes in the Methods section of your manuscript.

Reported on page number: There were no deviations from the study protocol

Did this study involve local collaborators that are residents of the country where the research was conducted or members of the community studied? If you do not have any authors from said communities, please provide an explanation for this below.

The first author is a resident of the country where the research was conducted. There were no other authors from the state or community where the study was conducted. This is because they did not contribute substantially to the design of the study. The first author is by qualification a medical professional and a Public Health physician who works within the Public Health Systems in the country where the research was conducted.

Everyone listed as an author should meet PLOS’ criteria for authorship and all individuals who meet these criteria should be included in the author byline, rather than the acknowledgements. Authorship criteria is based on the International Committee of Medical Journal Editors (ICMJE) Uniform Requirements for Manuscripts Submitted to Biomedical Journals - for further information please see here: <https://journals.plos.org/plosone/s/authorship>.

**Human subjects research (e.g. health research, medical research, cross-cultural psychology)**

Did you obtain written informed consent from a representative of the local community or region before the research took place? How did you establish who speaks for the community? Details of written informed consent obtained from study participants should be reported separately in the Methods section of your manuscript.

Yes. A letter of permission to conduct the study and visit the health facilities to conduct the research was granted by the Honourable Commissioner for Health of the Oyo State Ministry of Health. This is a key stakeholder in health who reports to the Governor of the state.

How did members of the local community provide input on the aims of the research investigation, its methodology, and its anticipated outcome(s)?

A focal group discussion had been earlier conducted with pregnant women resident in the study community on the women’s expectations during childbirth and their perceptions on respectful maternity care. This informed the need to now objectively measure what women experience during childbirth.

When engaging with the local community, how did you ensure that the informed consent documents and other materials could be understood by local stakeholders?

The informed consent and 15- item RMC scale for the postpartum interview were translated to Yoruba (as this was the predominant language of the women recruited and studied), then back translated into English to aid the research assistants. The research assistants recruited were also resident in the state where the research was conducted and speak Yoruba. They could explain the purpose of the research to the women to obtain their consent and also conduct the postpartum interview in the Yoruba language guided by the Yoruba translated version of the tool.

Will the findings of the research be made available in an understandable format to stakeholders in the community where the study was conducted (e.g. via a presentation, summary report, copies of publications, etc.)? Please provide details of how this will be achieved.

A brief presentation of the findings has been made at a dissemination meeting with four representatives from each of the nine study health facilities visited. We also produced a 1-page summary of the findings and gave to 17 key stakeholders and decision makers in the Oyo state Ministry of Health. Copies of this publication will also be shared with the Officers in charge of the health facility and these key stakeholders.

**Non-human subjects research using specimens/ animals collected as part of the study, or those housed in archival collections. Examples include archaeology, palaeontology, botany and zoology.**

Did the permission you obtained from a local authority to perform the study include an agreement on access to outputs and benefit sharing? This may include procedures to enable fair distribution of the benefits and resources arising from the research performed. Please include any details of Prior Informed Consent and Benefit Sharing Agreements obtained. These may be required by field-specific regulations, for example the Convention on Biological Diversity (CBD) and the associated Nagoya Protocol.

There are no Benefit Sharing Agreements in the informed consent and permission granted to conduct the study at the health facilities by the Honourable Commissioner for Health of the Oyo State Ministry of Health.

If the material used in your study was imported, please A) provide the year it was imported and B) indicate whether permits were obtained to import/export the materials used, C) provide details of any permits obtained. If this information is not available, please indicate this.

The study instruments were not imported. They were adapted standardised instruments used in other researches obtain from the literature. References have been made to the source of the study questionnaires. No equipment or study material was obtained from the literature.

If you used archival specimens, please state how the material used in your study was acquired by the institute it is held in and provide details of any permits obtained for the original excavations/ sample collection. If this information is not available, please indicate this.

There were no archival specimens used for this study

How was the potential cultural significance of the materials collected in your study to local communities considered in your research design? Were Indigenous peoples and/or local researchers and institutions involved with archaeological excavations / collection of specimens? If so, please provide a description of their involvement.

This is not applicable to this study

If your manuscript includes photographs of human remains, please indicate whether authors’ obtained permission from descendants or affiliated cultural communities to do so.

Our manuscript does not include photographs of human remains.
