# Supplementary material for: Directly observed and reported respectful maternity care received during childbirth in public health facilities, Ibadan Metropolis, Nigeria
Source: PLoS One. 2022 Oct 21;17(10):e0276346. doi: 10.1371/journal.pone.0276346 (PMC9586397; doi:10.1371/journal.pone.0276346)
Supplement: S3 File — (DOCX) [file pone.0276346.s003.docx]

**Supplement 3: Factors associated with reported RMC received at birth**

|  | **Simple Linear Regression Analysis** | | |  | **Multiple Regression Analysis** | | |
| --- | --- | --- | --- | --- | --- | --- | --- |
|  | **Crude Coeff.** | **LL – UL 95%CI** | **p-value** |  | **Adjusted Coeff.** | **LL – UL 95%CI** | **p-value** |
| **Age in years**  Youths (18–24 years)  Adults (25 – 35 years)  Adults (>35 years) | -0.354  Ref  0.858 | -4.642 - 3.934  -  -3.798 – 5.513 | 0.851  -  0.676 |  |  |  |  |
| **Education completed**  None/Primary education  Secondary education  Post-secondary education | Ref  2.224  2.913 | -  -2.162 – 6.610  -2.701 - 8.527 | -  0.270  0.259 |  |  |  |  |
| **Employment status**  Employed  Not employed | 1.415  Ref | -11.701 – 8.871  - | 0.754  - |  |  |  |  |
| **Monthly Income (usd) per 1000** | 4.0 | -12.0 – 20.0 | 0.601 |  |  |  |  |
| **Ethnicity**  Yoruba  Ibo | 4.761  Ref | -1.273 – 10.795  - | 0.104  - |  | 3.296  - | 0.089 – 6.503  - | **0.045**  - |
| ***Delivery health facility**  Facility 1  Facility 2  Facility 3  Facility 4  Facility 5  Facility 6  Facility 7  Facility 8  Facility 9 | -2.598  6.111  -7.664  2.715  -3.991  4.277  -3.118  1.680  2.589 | -6.177 – 0.982  3.540 – 8.682  -12.587 - -2.740  -0.665 – 6.094  -7.842 - -0.140  1.529 – 7.023  -10.904 – 4.667  -2.247 – 5.606  -0.220 – 5.398 | 0.154  **<0.001**  **0.002**  0.115  **0.042**  **0.002**  0.431  0.401  0.071 |  | -2.434  5.992  -7.577  2.263  -3.797  3.896  -2.827  1.540  2.944 | -2.584 - -2.283  5.758 – 6.225  -7.922 - -7.232  1.833 – 2.692  -4.008 - -3.587  3.405 - 4.387  -3.385 - -2.270  1.392 – 1.689  2.574 – 3.315 | **<0.001**  **<0.001**  **<0.001**  **<0.001**  **<0.001**  **<0.001** **<0.001**  **<0.001<0.001** |
| **Facility type**  Primary  Secondary | Ref  6.358 | -  0.480 – 12.237 | -  **0.038** |  |  |  |  |
| **Attending provider**  1 provider  2 providers | -0.530  Ref | -6.655 – 5.595  - | 0.844  - |  |  |  |  |
| **Provider familiarity**  Not familiar  Familiar (non-family)  Familiar (as family) | Ref  4.387  2.521 | -  0.909 – 7.865  -0.558 – 5.600 | -  **0.020**  0.094 |  | Ref  4.345  1.022 | -  0.140 – 8.551  -0.213 – 2.257 | -  **0.045**  0.091 |
| **Parity**  Primipara  Multipara | Ref  -0.311 | -  -3.833 – 3.211 | -  0.840 |  |  |  |  |
| **Booking status**  Booked  Un-booked | -0.997  Ref | -5.507 – 3.512  - | 0.617  - |  |  |  |  |
| **Presenting time-labour**  7:00am–18:59pm (DT)  19:00pm– 6:59am (NT) | Ref  -1.318 | -  -6.266 – 3.630 | -  0.549 |  |  |  |  |
| **Constant** |  |  |  |  | 70.634 | 67.612 – 73.657 | **<0.001** |
|  |  |  |  |  | *n=269; R^2^= 0.090;* ***p=0.0002*** | | |

**Significant p values in bold. DT- daytime, NT- Night time. *These results are shown as contrasts against the mean**
